# P-proteins in *Arabidopsis* are heteromeric structures involved in rapid sieve tube sealing

**DOI:** 10.3389/fpls.2013.00225

**Published:** 2013-07-03

**Authors:** Stephan B. Jekat, Antonia M. Ernst, Andreas von Bohl, Sascia Zielonka, Richard M. Twyman, Gundula A. Noll, Dirk Prüfer

**Affiliations:** ^1^Fraunhofer Institute for Molecular Biology and Applied Ecology, MünsterGermany; ^2^Institute of Plant Biology and Biotechnology, University of Münster, MünsterGermany; ^3^Institute for Molecular Biotechnology, Rheinisch-Westfaelische Technische Hochschule Aachen UniversityAachen, Germany; ^4^Twyman Research Management LtdYork, UK

**Keywords:** sieve element occlusion, phloem, translocation, wound sealing, exudation, forisome

## Abstract

Structural phloem proteins (P-proteins) are characteristic components of the sieve elements in all dicotyledonous and many monocotyledonous angiosperms. Tobacco P-proteins were recently confirmed to be encoded by the widespread *sieve element occlusion (SEO)* gene family, and tobacco SEO proteins were shown to be directly involved in sieve tube sealing thus preventing the loss of photosynthate. Analysis of the two *Arabidopsis* SEO proteins (AtSEOa and AtSEOb) indicated that the corresponding P-protein subunits do not act in a redundant manner. However, there are still pending questions regarding the interaction properties and specific functions of AtSEOa and AtSEOb as well as the general function of structural P-proteins in *Arabidopsis*. In this study, we characterized the *Arabidopsis* P-proteins in more detail. We used *in planta* bimolecular fluorescence complementation assays to confirm the predicted heteromeric interactions between AtSEOa and AtSEOb. *Arabidopsis* mutants depleted for one or both AtSEO proteins lacked the typical P-protein structures normally found in sieve elements, underlining the identity of AtSEO proteins as P-proteins and furthermore providing the means to determine the role of *Arabidopsis* P-proteins in sieve tube sealing. We therefore developed an assay based on phloem exudation. Mutants with reduced AtSEO expression levels lost twice as much photosynthate following injury as comparable wild-type plants, confirming that *Arabidopsis* P-proteins are indeed involved in sieve tube sealing.

## INTRODUCTION

The complex phloem system of higher plants distributes photoassimilates and signaling molecules throughout the plant body. The conducting sieve tubes that accomplish this long-distance transport comprise sieve elements connected end-to-end with intervening perforated sieve plates to promote efficient mass flow. Angiosperm sieve elements are characterized by abundant structural phloem proteins (P-proteins; Cronshaw and Esau, 1967), the identity and function of which has been debated for decades.

P-proteins of the fascicular phloem are today known to be encoded by members of the widespread *sieve element occlusion* (*SEO*) gene family (Pélissier et al., 2008; Rüping et al., 2010; Ernst et al., 2011,^2012^; Froelich et al., 2011). *SEO-F* (*sieve element occlusion by forisomes*) members of the family were first described in Fabaceae and shown to encode forisome components (Noll et al., 2007; Pélissier et al., 2008; Müller et al., 2010; Rüping et al., 2010). Forisomes are spindle-shaped P-proteins that can undergo a reversible conformational change to occlude sieve tubes (Knoblauch et al., 2001,^2003^). The subsequent characterization of SEO proteins in non-Fabaceae plants confirmed their proposed role as conventional P-proteins in angiosperms (Froelich et al., 2011; Anstead et al., 2012; Ernst et al., 2012).

P-proteins subunits are synthesized in immature, nucleated sieve elements (Cronshaw and Esau, 1967,^1968^; Esau and Cronshaw, 1967; Noll et al., 2007,^2009^; Rüping et al., 2010; Bucsenez et al., 2012; Ernst et al., 2012) and initially assemble as large protein bodies (Steer and Newcomb, 1969). Toward the end of sieve element maturation, these bodies disperse into filaments that relocate to the cell periphery and ultimately form a meshed layer in functional sieve tubes (Evert et al., 1973; Knoblauch and van Bel, 1998; Froelich et al., 2011). Following injury, P-protein structures are dislodged from their parietal position and translocate toward the downstream sieve plate where they accumulate as rather large viscous plugs (Anderson and Cronshaw, 1970). The long-discussed ability of these P-protein plugs to halt or at least reduce photoassimilate translocation was recently demonstrated in tobacco plants lacking normal amounts of P-proteins (Ernst et al., 2012), but it is unclear whether the same principles apply in *Arabidopsis* (Froelich et al., 2011). This raises the interesting possibility that P-proteins play varying roles in the sieve tube sealing process in different plant species.

It is also unclear whether there is redundancy among the SEO proteins in some species. The number of *SEO* genes identified in different plant species ranges from three (including one pseudogene) in *Arabidopsis* to 26 (including seven pseudogenes) in soybean (Rüping et al., 2010; Ernst et al., 2011) and the conserved sequences and expression profiles suggest potential functional redundancy among the corresponding proteins (Lynch and Conery, 2000). This issue was recently addressed in *Arabidopsis*, where transfer DNA (T-DNA) insertion mutants lacking either AtSEOa or AtSEOb did not accumulate the typical P-protein filaments in sieve elements, although the remaining *AtSEO* gene was expressed normally (Anstead et al., 2012). This was confirmed by microscopy in the same mutant lines complemented with a green fluorescent protein (GFP)-tagged version of the missing AtSEO protein. These data indicate that AtSEOa and AtSEOb are likely to be structural phloem filament proteins that are jointly required for filament formation. However, the fundamental ability to form heteromers could not be confirmed.

We therefore set out to characterize the role of SEO proteins in *Arabidopsis* in more detail. The anticipated heteromeric interaction between AtSEOa and AtSEOb was confirmed by bimolecular fluorescence complementation (BiFC) studies *in planta*. Furthermore, the generation and analysis of mutants with limited expression of both *AtSEO* genes confirmed the hypothesis that AtSEOa and AtSEOb are *Arabidopsis* P-proteins because the typical P-protein structures could accordingly not be detected in these plants by transmission electron microscopy (TEM). The P-protein-depleted plants also allowed us to examine the functional role of structural P-proteins in *Arabidopsis*. We found that the mutants lost twice as much photosynthate as comparable wild-type plants following injury, providing the first direct evidence that P-proteins are required for rapid phloem wound sealing in the model plant *Arabidopsis*.

## MATERIALS AND METHODS

### *Arabidopsis* T-DNA INSERTION MUTANTS

Seeds of T-DNA insertion mutants for the genes *AtSEOa* (*At3g01670*) and *AtSEOb* (*At3g01680*) were obtained from the European *Arabidopsis* Stock Centre (NASC). Homozygous plants were identified from the lines SALK_148614C (Δ*AtSEOa*) and SM_3_34780 (Δ*AtSEOb*), which were used for further analysis. All mutants are in a Columbia (Col-0) background.

### PLANT GROWTH CONDITIONS

Plants were typically grown in phytochambers at 23/18°C, 40% relative humidity and under long-day conditions (16/8-h light/dark period). To increase the leaf size for exudation analysis, the corresponding lines were cultivated under short-day conditions (8/16-h light/dark period).

### AGROINFILTRATION OF *Nicotiana* benthamiana

The interaction characteristics of AtSEOa and AtSEOb were analyzed by BiFC in a plant background (Bracha-Drori et al., 2004; Walter et al., 2004). The coding sequences of both genes were amplified with and without translational stop codons using primer combinations 5′-AGA TCATGA AGA TGG CCC AAC GCT TTC AAT-3′ and 5′-ACA TCTAGA TTA CTC AAG GCA GCA TTG GT-3′ or 5′-ACA TCTAGA CCC TCA AGG CAG CAT TGG-3′ (*AtSEOa*) and 5′-AGA TCATGA AGA TGG AGT CGC TGA TCA AG-3′ and 5′-ACA TCTAGA TTA GAA GTT GTA GTT CTC GTC-3′ or 5′-ACA TCTAGA CCG AAG TTG TAG TTC TCG TC-3′ (*AtSEOb*). After digestion with *Bsp*HI/*Xba*I, the gene fragments were ligated into the *Nco*I/*Xba*I sites of entry vector pENTR4 (Invitrogen). The recombinant entry vectors were used to insert the *AtSEO* sequences into GATEWAY-compatible pBatTL vectors, mediated by LR Clonase (Invitrogen). The pBatTL vectors contained reporter sequences encoding split variants of the monomeric red fluorescent protein mRFP1-Q66T (Jach et al., 2006) resulting in eight different constructs (pBatTL*AtSEOa/b:NmRFP*, pBatTL*AtSEOa/b:CmRFP*, pBatTL*NmRFP:AtSEOa/b*, and pBatTL*CmRFP:AtSEOa/b*). A monomeric version of Emerald (mEmerald) was used as a control by converting the phenylalanine residue at position 223 to arginine by polymerase chain reaction (PCR) mutagenesis using primers 5′-AGA ACCATGGGT AAA GGA GAA G-3′ and 5′-AGA ACTCGAGTG TTT GTA TAG TTC ATC CAT GCC ATG TGT AAT CCC AGC AGC TGT TAC TCT CTC AAG AAG GAC CAT GTG-3′ with vector pGJ2628 as the template (kindly provided by Dr. Guido Jach). The mEmerald fragment was digested with *Nco*I/*Xho*I and inserted into vector pENTR4, which was again used to insert the gene into the corresponding pBatTL vectors resulting in constructs pBatTL*mEmerald:NmRFP* and pBatTL*mEmerald:CmRFP*.

For transient expression in *N. benthamiana* epidermal cells, all BiFC constructs were introduced into *Agrobacterium tumefaciens* strain GV31.01 pMP90. Leaves from 4-week-old plants were infiltrated simultaneously with *A. tumefaciens* GV31.01 pMP90 (carrying the pBatTL vectors) and *A. tumefaciens* C58C1 (carrying the pCH32 helper plasmid encoding *Tomato bushy stunt virus *RNA silencing repressor p19; Voinnet et al., 2003). Infiltrated leaf discs were punched out after 3 days and analyzed by confocal laser scanning microscopy (CLSM).

### CLSM IMAGING

*N. benthamiana* leaf discs were analyzed by CLSM using a Leica TCS SP5X microscope with excitation/emission wavelengths of 543/570–630 nm for the detection of mRFP and 488/500–580 nm for the detection of mEmerald.

### GENERATION OF AtSEO KNOCKOUT/KNOCKDOWN LINES

To obtain *Arabidopsis* plants with reduced levels of both AtSEO proteins, RNAi vectors producing AtSEOa or AtSEOb hairpin (hp) RNAs were generated and used to transform the homozygous T-DNA insertion mutants (Δ*AtSEOa* orΔ*AtSEOb*) described above. To ensure strong transgene expression in sieve elements, the *Cauliflower mosaic virus* (CaMV) 35S promoter from the RNAi vector pHANNIBAL (Wesley et al., 2001; kindly provided by CSIRO Plant Industry) was removed by digestion with *Sac*I/*Eco*RI (including Klenow treatment) or *Sac*I/*Xho*I, and replaced with 997-bp promoter fragments designated P*AtSEOa* and P*AtSEOb*, amplified from *Arabidopsis* Col-0 genomic DNA. The P*AtSEOa Sma*I/*Eco*RI primer combination was 5′-AGA CCCGGG GTC GAGCGGCCGCTC AGC CGG AGA TCA TCC-3′ and 5′-AGA GAATTC ATT GGC GAG GTT GAG AG-3′ to generate P**_AtSEOa_/pHANNIBAL. The P*AtSEOb Sac*I/*Xho*I primer combination was 5′-AGA GAGCTC GTC GAGCGGCCGCAA AAC ATG CAT AGA ATA AAC C-3′ and 5′-AGA CTCGAG TCT TGG TTC AGT TTG CTT TTG-3′ to generate P**_AtSEOb_/pHANNIBAL. In each case, the *Not*I site was introduced for a subsequent cloning step. 400-bp fragments with different restriction sites were then amplified from complementary DNA (cDNA) for *AtSEOa* (primer combinations 5′-AGA GAATTC CAA GAT CCG CCG AGC-3′/5′-AGA GGTACC TCA AAG AAG ATC ATG TCT GG-3′ and 5′-AGA GGATCC TCA AAG AAG ATC ATG TCT GG-3′/5′-AGA TCTAGA CAA GAT CCG CCG AGC-3′) and *AtSEOb* (primer combinations 5′-AGAGGTACC CTG TTC TGC ACA GGG AC-3′/5′-AGA GGTACC CTC GCG AAG TCC AAG TG-3′ and 5′-AGA AAG CTT CTG TTC TGC ACA GGG AC-3′/5′-AGA TCT AGA CTC GCG AAG TCC AAG TG-3′). The gene fragments were inserted into the *Eco*RI/*Kpn*I and *Bam*HI/*Xba*I sites (*AtSEOa*) or *Kpn*I and *Hind*III/*Xba*I sites (*AtSEOb*) of the modified pHANNIBAL vectors to obtain P**_AtSEOa_hp*AtSEOa*/pHANNIBAL and P**_AtSEO__b_hp*AtSEOb*/pHANNIBAL. The hpRNA cassettes were isolated by digestion with *Not*I and, after Klenow treatment, inserted into the *Sma*I sites of binary plant transformation vectors pBIN19 (Bevan, 1984) or pLab12.1 (Post et al., 2012), resulting in the final constructs pBP**_AtSEOa_hp*AtSEOa* and pLP**_AtSEOb_hp*AtSEOb*.

### STABLE PLANT TRANSFORMATION

All binary vectors used for stable plant transformation were introduced into *A. tumefaciens *strain LBA4404 by electroporation. *Arabidopsis* plants were transformed using the floral dip method (Clough and Bent, 1998).

### REVERSE TRANSCRIPTASE PCR

*AtSEO* gene expression in the mutant lines was analyzed by isolating total RNA from young leaves using the NucleoSpin RNA Plant Kit (Macherey-Nagel) followed by reverse transcriptase (RT)-PCR. Superscript II (Invitrogen) was used for reverse transcription, followed by RNA digestion (RNaseH; New England Biolabs). Intron-spanning gene fragments were amplified using primer combinations 5′-TGA TGT CAC ATC ACT TCT CTC CG-3′/5′-TGC CAT GCT TCT GTG TAG AG-3′ (*AtSEOa*) and 5′-ATG GAG TCG CTG ATC AAG-3′/5′-CAG TGA TCA TGT TGA TCT GAG-3′ (*AtSEOb*). The *Arabidopsis* actin gene *ACT2* (accession number U41998) was amplified as an internal control using primers 5′-CCT CAT CAT ACT CGG CCT TGG AG-3′ and 5′-GTA AGA GAC ATC AAG GAG AAG CTC TC-3′.

### TEM

The ultrastructure of the *Arabidopsis* sieve elements was analyzed by cutting small stem segments, bisecting them longitudinally, fixing the explants in 2% (vol/vol) glutaraldehyde and 3.5% (wt/vol) sucrose for 3 h and incubating them in 1% (vol/vol) osmium tetroxide for 2 h. Samples were dehydrated in ethanol and embedded in LR White (Sigma). Ultrathin sections were stained for 30 min in 2% (wt/vol) uranyl acetate and 3 min in 4% (wt/vol) lead citrate. The sections were examined with a Zeiss EM900 microscope fitted with an SIS Morada digital camera.

### EXUDATION ANALYSIS

*Arabidopsis* plants (wild-type, *n* = 38; Δ*AtSEOa*, *n* = 44; Δ*AtSEOb*, *n* = 28; Δ*AtSEOa*/↓*AtSEOb* lines I, *n* = 30; II, *n* = 52; III, *n* = 54; Δ*AtSEOb*/↓*AtSEOa* lines I, *n* = 40; II, *n* = 44; III, *n* = 54) were grown under short-day conditions for 9 weeks. Fifteen mature leaves were cut from every plant included in the analysis with a razor blade, jointly placed in one vial [containing 3 mL 1 mM MES (2-(*N*-morpholino)ethanesulfonic acid), pH 7] and exuded for 10 min. Exudation experiments were carried out consecutively for 2 days between zeitgeber times 2 and 8 with plants of all lines used in an alternating manner. After exudation, samples were frozen in liquid nitrogen and lyophilized. Freeze-dried samples of two plants were combined and dissolved in 120 μL distilled water, and 50 μL of the solution was then used to determine the quantity of D-glucose and sucrose using the sucrose/D-glucose/D-fructose kit (Roche) as described by Ernst et al. (2012). To determine the total sucrose content of total leaves, these were ground under liquid nitrogen, and the extraction and quantification of sugars was carried out using the sucrose/D-glucose/D-fructose kit (Roche) according to the manufacturer’s instructions.

## RESULTS

### AtSEOa AND AtSEOb FORM HETEROMERS IN A PLANT BACKGROUND

**FIGURE 1:**
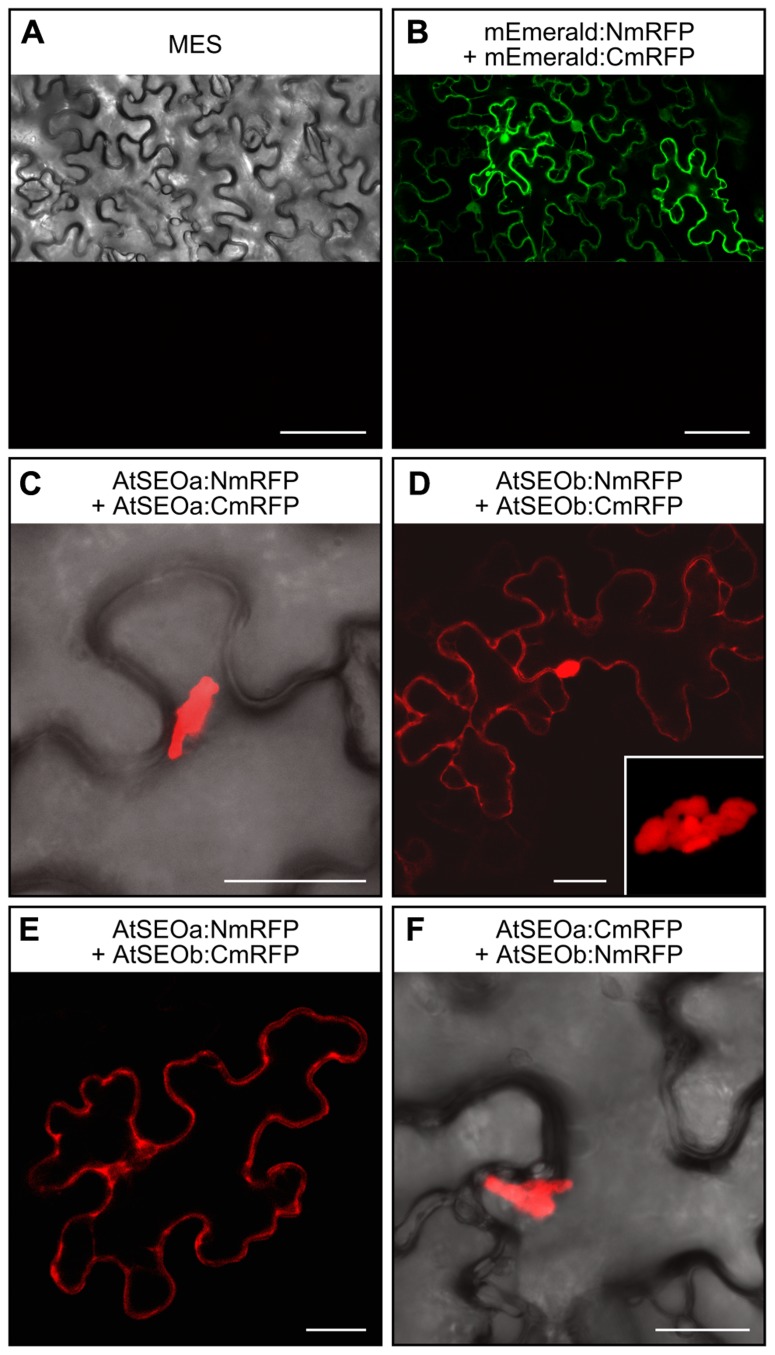
**BiFC-based interaction studies in *N. benthamiana* epidermal cells.** **(A)** No red fluorescence was detected at 3 dpi in *N. benthamiana* leaves infiltrated with MES buffer alone. **(B)** When mEmerald was introduced into the split mRFP vector system, green fluorescence confirmed the activity of the expression cassettes. No red fluorescence could be detected, excluding the appearance of false positive signals due to non-specific reporter fragment contact. **(C)** Homomeric interactions among AtSEOa subunits always resulted in red fluorescent structural complexes. **(D)** Homomeric interactions among AtSEOb subunits were detected as either cytoplasmic fluorescence or as fluorescent complexes (the inset image provides a detailed view of the complex). Fluorescence indicating heteromeric interactions between AtSEOa and AtSEOb was either evenly distributed in the cytoplasm **(E)** or derived from structural complexes **(F)**. Scale bars = 75 μm **(A,B)** and 25 μm **(C,D,E,F)**.

AtSEOa and AtSEOb were assumed to be non-redundant proteins that undergo heteromeric interactions to form P-protein structures because the analysis of AtSEO complementation lines by microscopy showed typical filaments only when both proteins were expressed (Anstead et al., 2012). However, these data could not be confirmed in yeast two-hybrid experiments, which showed that both AtSEOa and AtSEOb could readily form homodimers but not heterodimers. To resolve this apparent inconsistency and to check whether heteromeric interaction might occur in higher-order structures, we analyzed the interaction behavior of AtSEOa and AtSEOb in a plant background using BiFC. To reduce any potential impact of the reporter fragments, we fused both split variants of the monomeric red fluorescent protein mRFP-Q66T (Jach et al., 2006) to the N- and C-terminus of AtSEOa and AtSEOb, respectively, resulting in eight different constructs (pBatTL*AtSEOa/b:NmRFP*, pBatTL*AtSEOa/b:CmRFP*, pBatTL*NmRFP:AtSEOa/b*, and pBatTL*CmRFP:AtSEOa/b*). These were introduced into *N. benthamiana* leaves by agroinfiltration using all possible heteromeric combinations along with appropriate controls (**Figure 1**).

The infiltration of MES buffer alone did not provoke any defense reactions that might cause red fluorescence in the epidermal cells (**Figure 1A**). Furthermore, to exclude reporter fluorescence caused by the non-specific interaction of the mRFP split fragments, we fused these to the coding sequence of the non-interacting protein mEmerald. Leaves infiltrated with these control constructs showed green fluorescence confirming that the mEmerald fusions were efficiently expressed (upper part of **Figure 1B**), whereas no mRFP fluorescence could be detected (lower part of **Figure 1B**). Homomeric interaction events could be detected for both AtSEOa and AtSEOb in all tested combinations. AtSEOa homomeric interactions always resulted in rather large fluorescent complexes (**Figure 1C**, showing the combination AtSEOa:NmRFP + AtSEOa:CmRFP as an example), but AtSEOb homomeric interaction often yielded cytoplasmic fluorescence, although some combinations also yielded complexes (**Figure 1D**, showing the combination AtSEOb:NmRFP + AtSEOb:CmRFP as an example). Most interestingly, the *in planta *BiFC approach showed definite interaction between AtSEOa and AtSEOb (**Figures 1E,F**). Depending on the tag position and the reporter protein variant, fluorescence was either evenly distributed in the cytoplasm (**Figure 1E**, showing the combination AtSEOa:NmRFP + AtSEOb:CmRFP as an example) or, more often, restricted to large protein complexes (Figure 1F, showing the combination AtSEOa:CmRFP + AtSEOb:NmRFP as an example). These experiments confirmed that AtSEOa and AtSEOb can indeed interact in a heteromeric manner.

### AtSEO KNOCKOUT/KNOCKDOWN PLANTS LACKING STRUCTURAL P-PROTEINS SHOW NO DISTINCT MORPHOLOGICAL PHENOTYPE

**FIGURE 2:**
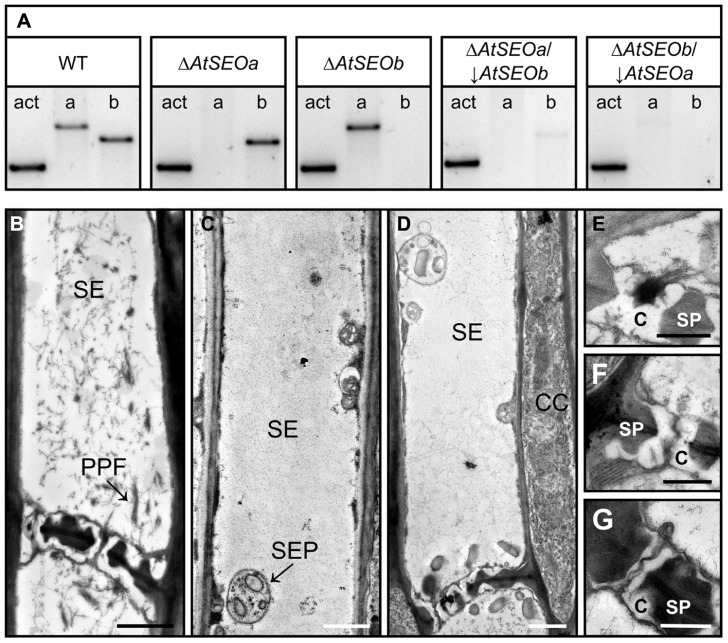
**Structural analysis of AtSEO-depleted *Arabidopsis* mutants.(A)** *AtSEO* single-knockout plants (Δ*AtSEOa* andΔ*AtSEOb*) were used to generate knockout/knockdown plants impaired in the expression of both *AtSEO* genes (Δ*AtSEOa*/↓*AtSEOb* and Δ*AtSEOb*/↓*AtSEOa*). **(B)** TEM showing sieve elements from wild-type *Arabidopsis* plants characterized by abundant P-protein filaments clearly occluding sieve pores **(E)**. In contrast, the sieve elements of mutants Δ*AtSEOa*/↓*AtSEOb ***(C,F)** and Δ*AtSEOb*/↓*AtSEOa* **(D,G)** were free of P-protein and no filaments were observed in the sieve pores **(F,G)**. C, callose; CC, companion cell; PPF, P-protein filaments; SE, sieve element; SEP, sieve element plastid; SP, sieve plate. Scale bars = 1 μm **(B,C,D)** and 0.5 μm **(E,F,G)**.

AtSEOa and AtSEOb are thought to be non-redundant proteins and single knockout mutants lacking either AtSEOa or AtSEOb are known to lack the typical P-protein filaments (Anstead et al., 2012). However, because the precise functions of AtSEOa and AtSEOb in P-protein formation are still unknown, we next set out to generate plants completely lacking AtSEO proteins for further analysis. Therefore, the T-DNA insertion mutants Δ*AtSEOa* and Δ*AtSEOb *were supertransformed with hpRNA cassettes specific for the remaining functional *AtSEO* gene, resulting in knockout/knockdown plant lines designated Δ*AtSEOa*/↓*AtSEOb* and Δ*AtSEOb*/↓*AtSEOa*. The efficiency of the knockout/knockdown combination was analyzed at the messenger RNA (mRNA) level by RT-PCR using intron-spanning primers. **Figure 2A** shows the expression profiles of *AtSEOa* and *AtSEOb* in the young leaves of wild-type *Arabidopsis* plants, T-DNA insertion mutants and the RNAi lines, confirming the knockout of *AtSEOa* and *AtSEOb* in Δ*AtSEOa* and Δ*AtSEOb *and the efficient silencing of the non-interrupted remaining *AtSEO* gene in Δ*AtSEOa*/↓*AtSEOb* and Δ*AtSEOb*/↓*AtSEOa*. Minimal residual expression of the silenced *AtSEO* genes was detected in the knockout/knockdown lines (**Figure 2A**) and we were unable to confirm silencing directly at the protein level because no antibodies are available for the specific detection of AtSEOa or AtSEOb.

Next, we used TEM to investigate the structural properties of sieve elements. Therefore, stem sections from 5-week-old plants (wild-type, Δ*AtSEOa*, Δ*AtSEOb*, Δ*AtSEOa*/↓*AtSEOb*, and Δ*AtSEOb*/↓*AtSEOa*) were prepared as described above. Sieve elements of *Arabidopsis* wild-type plants were always characterized by the abundance of P-protein filaments (**Figure 2B**). Because of the wounding caused by explant preparation, P-proteins were usually detected in the dispersed state with filaments detached from their parietal positions and clearly occluding the sieve pores (**Figure 2E**). In agreement with previous studies, we found that the T-DNA insertion mutants Δ*AtSEOa* and Δ*AtSEOb* (only impaired in expression of one single *AtSEO* gene) were also unable to form typical P-protein structures, confirming that both AtSEOa and AtSEOb are needed to form the filaments (Froelich et al., 2011; Anstead et al., 2012). The knockout/knockdown lines with only residual *AtSEO *gene expression (Δ*AtSEOa*/↓*AtSEOb* and Δ*AtSEOb*/↓*AtSEOa*) also completely lacked P-protein-like structures in the sieve elements (Figures 2C,D) and no filaments accumulated at the sieve plates occluding sieve pores (Figures 2F,G). Sieve element morphology was not affected by the lack of structural P-proteins.

Plants of all lines were grown under standard conditions for several weeks and none of the mutants showed any obvious phenotype differing from wild-type plants. The availability of these plants therefore enabled us to analyze the long-discussed functional role of *Arabidopsis* P-proteins in sieve tube sealing.

### *Arabidopsis* PLANTS LACKING P-PROTEINS SHOW ENHANCED BLEEDING FOLLOWING INJURY

Although P-proteins are known to disperse from the cell periphery and accumulate on downstream sieve plates after injury, their direct role in sieve tube sealing has long been subject to debate. The first direct evidence for their capacity to prevent phloem translocation was provided recently when tobacco mutants lacking P-proteins were tested in exudation assays, showing that the depleted plants lost on average nine times more photoassimilate than corresponding wild-type plants (Ernst et al., 2012). However, the function of P-proteins in *Arabidopsis* has been questioned based on the microscopic analysis of plants expressing AtSEO-yellow fluorescent protein (YFP) fusion constructs (Froelich et al., 2011). To address this discrepancy, we developed an exudation assay to compare the loss of photoassimilate in wild-type *Arabidopsis* plants and P-protein-depleted mutants following injury.

**FIGURE 3:**
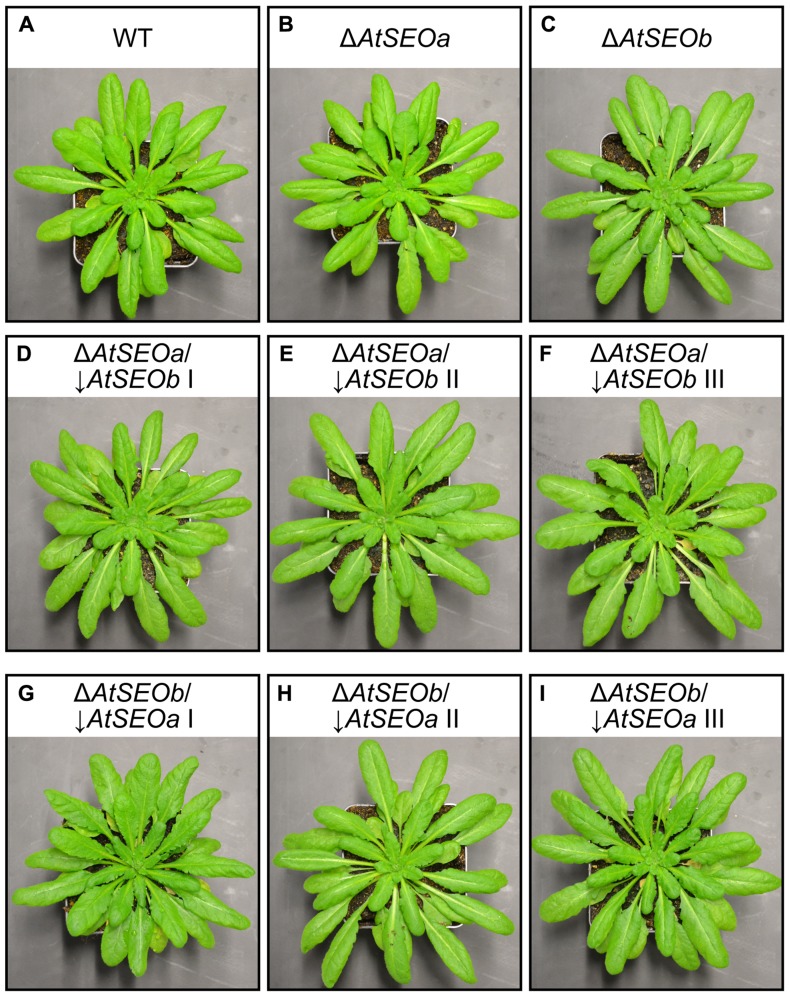
***Arabidopsis* plants used for exudation analysis.** *Arabidopsis* wild-type **(A)**, single-knockout Δ*AtSEOa* **(B)** and Δ*AtSEOb***(C)** and knockout/knockdown plants Δ*AtSEOa*/↓*AtSEOb*, lines I–III **(D,E,F)**, and Δ*AtSEOb*/↓*AtSEOa*,lines I–III **(G,H,I)**, did not show any obvious morphological phenotype prior to the exudation assay. All plants were grown under short-day conditions to increase total leaf size.

We therefore grew wild-type *Arabidopsis* plants, single-knockout mutants Δ*AtSEOa* and Δ*AtSEOb* and corresponding knockout/knockdown plants (Δ*AtSEOa*/↓*AtSEOb *and Δ*AtSEOb*/↓*AtSEOa*, lines I, II, and III). To increase the average leaf size, all plants were cultivated under short-day conditions and again no significant morphological phenotype was visible when exudation experiments were carried out at the age of 9 weeks (**Figure 3**). In *Arabidopsis*, most of the sugar is translocated as sucrose (Haritatos et al., 2000). Therefore, the amount of sucrose lost after injury should mirror the efficiency of sieve tube sealing.

**FIGURE 4:**
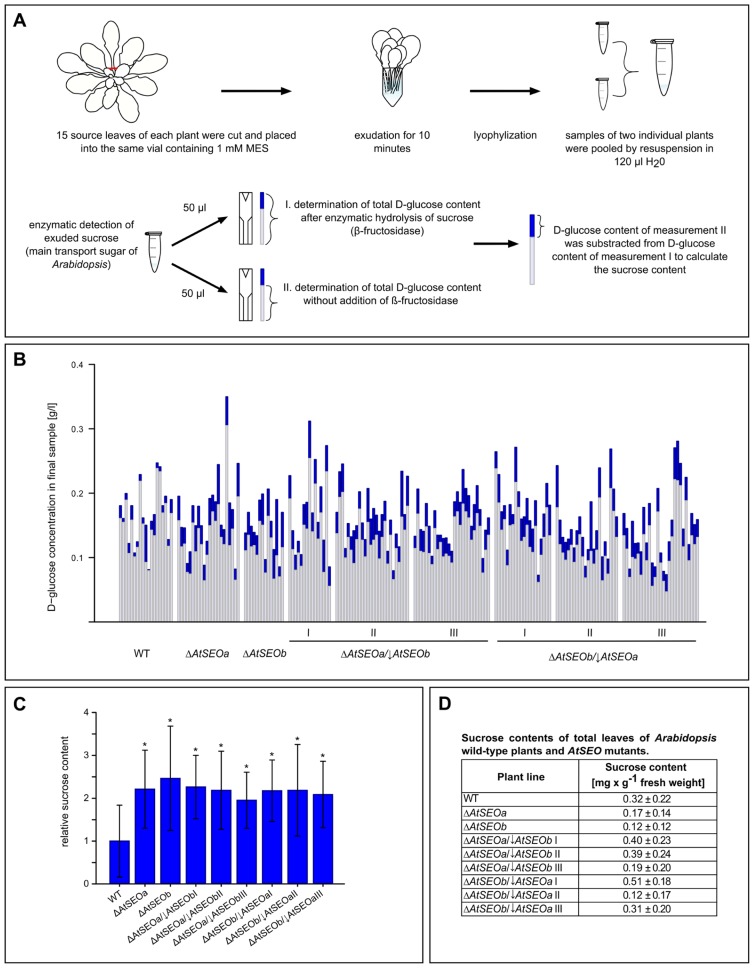
**Analysis of exudation rates in wild-type *Arabidopsis* plants, single AtSEO knockout mutants and AtSEO knockout/knockdown mutants.** **(A)** Schematic overview of the exudation studies. **(B)** The sucrose content of exudates from wild-type plants (WT) and mutants impaired in the expression of either one (Δ*AtSEOa* and Δ*AtSEOb*) or both *AtSEO* genes (Δ*AtSEOa*/↓*AtSEOb* and Δ*AtSEOb*/↓*AtSEOa*,lines I–III, respectively) was calculated from the difference in D-glucose concentration before (light blue parts of bars) and after (total bars) the enzymatic conversion of sucrose. **(C)** Summary of the exudation assays. The mean value of sucrose exuded from wild-type plants was set as 1. The results represent means ± SD; *n*_WT_ = 19, *n*_Δ_*AtSEOa* = 22, *n*_Δ_*AtSEOb* = 14, *n*_Δ_*AtSEOa*/↓*AtSEO_blineI_* = 15, *n*_Δ_*AtSEOa*/↓*AtSEO_blineII_* = 26, *n*_Δ_*AtSEOa*/↓*AtSEO_blineIII_* = 27, *n*_Δ_*AtSEOb*/↓*AtSEO_alineI_* = 20, *n*_Δ_*AtSEOb*/↓*AtSEO_alineII_* = 22, *n*_Δ_*AtSEOb*/↓*AtSEO_alineIII_* = 27; *P ≤ 0.001, Mann–Whitney *u* test. **(D) **Total leaf controls: total sugars were extracted from ground tissue and sucrose concentrations were determined as described above. The results represent means ± SD; *n*_WT_ = 8; *n*_Δ*AtSEOa*_ = *n*_Δ*AtSEOb*_ = *n*_Δ*AtSEOa*_/_↓*AtSEObI-III*_ = *n*_Δ*AtSEOb*/↓*AtSEOaI-III*_ = 4.

An experimental overview of the exudation assay is provided in **Figure 4A**. We removed 15 mature leaves from each assayed plant and pooled the exudates in one vial. We limited exudation to 10 min, thus measuring the rapid sealing activity of P-proteins while avoiding the onset of callose deposition, which promotes the long-term sealing of sieve pores (van Bel, 2003; Xie et al., 2011). After exudation, the samples were lyophilized and the freeze-dried contents from two vials were redissolved and combined, therefore representing the total exudates of 30 leaves derived from two individual plants of the same line. We then determined the D-glucose concentration of these final samples before and after the enzymatic hydrolysis of sucrose using the sucrose/D-glucose/D-fructose kit (Roche), and sucrose concentrations were deduced from the difference between the D-glucose concentrations before and after enzymatic inversion (Figures 4A,B). Remarkably, all the mutant lines tested in the exudation experiments showed a similar enhanced loss of photoassimilate compared to wild-type plants (**Figure 4C**). Mutant plants lacking one *AtSEO* gene and knockout/knockdown plants impaired in expression of both *AtSEO *genes lost on average twice as much sucrose as wild-type plants. To exclude the possibility that the differences in sucrose content might reflect the presence of more sucrose in the leaves of the mutant plants, we prepared total leaf extracts from all plant lines included in the assay and determined the sucrose concentrations as described above. These measurements confirmed that the sucrose contents in leaves of mutants were not generally increased (**Figure 4D**) and that the enhanced loss of transport sugars should therefore be a direct consequence of diminished P-protein contents. Our experiments therefore show that the lack of P-protein reduces the wound-sealing capacity of *Arabidopsis* plants. Furthermore, comparable exudation rates of the single-knockout and knockout/knockdown mutants confirm that both AtSEOa and AtSEOb are required to form functional P-protein structures in *Arabidopsis* sieve elements.

## DISCUSSION

### NEW INSIGHTS INTO AtSEO INTERACTIONS

The analysis of AtSEO complementation lines clearly indicated that both AtSEOa and AtSEOb are required to form the typical P-protein structures in sieve elements, because filaments could only be detected when both proteins were present (Anstead et al., 2012). This would almost certainly require some form of heteromeric interaction between the subunits, although heterodimerization events have not been detected in previous yeast two-hybrid experiments. We therefore carried out interaction studies based on BiFC in a plant background. The infiltration of homomeric combinations led to the formation of rather large complexes of AtSEOa protein, whereas homomeric interaction events for AtSEOb often resulted in cytoplasmic fluorescence but also yielded similar complexes. Our experiments therefore confirmed the homomeric interaction potential of both AtSEOa and AtSEOb (Anstead et al., 2012) and established that the interactions also occur *in planta*. The combinatorial expression of AtSEOa and AtSEOb fusions finally confirmed the predicted ability of the proteins to interact in a heteromeric manner. These combinations yielded complexes as well as evenly distributed cytoplasmic fluorescence. Because heteromers could be demonstrated in plants but not in the yeast two-hybrid system, it is possible that the interaction between AtSEOa and AtSEOb involves higher-order structures, perhaps requiring the initial assembly of homomeric multimers, although this will need to be investigated in future experiments.

AtSEOa and AtSEOb are both required to form typical P-protein filaments in *Arabidopsis*, indicating that the proteins are not functionally redundant, although their functions may overlap (Anstead et al., 2012). Our investigation of homomeric interactions revealed different reporter signals for each protein: AtSEOa formed complexes, whereas AtSEOb formed complexes but was also detected as uniform cytoplasmic fluorescence. Such distinct behavior may provide evidence that AtSEOa and AtSEOb have different roles in the formation of P-proteins, but the data should not be over-interpreted because the interaction was investigated in a non-phloem-cell background under the control of a non-native promoter. The structures observed during the interaction experiments were also clearly influenced by the position and/or size of the fused split mRFP fragments.

Analogous investigations focusing on tobacco SEO proteins also demonstrated a strong potential for interaction (Ernst et al., 2012; Jekat et al., 2012). Further comparative analysis of the SEO proteins from different species could provide insight into the detailed mode of SEO interaction and polymerization, and the relevant motifs that allow SEO proteins to form multimers. However, *Arabidopsis* is currently the only angiosperm known to possess just two expressed *SEO* genes, whereas, e.g., 19 *SEO* genes (including *SEO-F* genes) are expressed in *Glycine max* (Rüping et al., 2010), suggesting there may be species-dependent functional differences among these proteins.

### AtSEO PROTEINS ARE INVOLVED IN SIEVE TUBE SEALING

The involvement of P-proteins in rapid sieve tube sealing in response to phloem wounding is a matter of debate. Phloem explants normally contain P-proteins in the wounding state with dispersed filaments accumulating at sieve plates and clearly occluding pores, which is why the structural components were proposed to be involved in sieve tube sealing (Cronshaw, 1981; Will and van Bel, 2006; Furch et al., 2010). The first direct evidence that P-proteins can indeed affect translocation was provided by the analysis of tobacco mutants depleted for SEO proteins, which lost on average nine times more photosynthate than wild-type plants upon injury (Ernst et al., 2012). However, microscopic analysis of AtSEO-YFP fusion proteins expressed in *Arabidopsis* sieve elements revealed slow movement of the resulting protein plugs through sieve plates, leading to the hypothesis that AtSEO proteins might not be able to affect transport through sieve tubes (Froelich et al., 2011). We therefore developed an *Arabidopsis* exudation assay to measure the direct loss of photosynthate after wounding.

The generation of AtSEO knockout/knockdown mutants lacking P-protein filaments in the sieve elements (but otherwise showing no significant morphological differences to wild-type plants) allowed us to investigate the function of *Arabidopsis* P-proteins following injury. Our results clearly showed that *Arabidopsis* P-proteins are required for sieve tube sealing because mutants lacking P-protein structures lost significantly greater amounts of transport sugars after wounding than corresponding wild-type plants. Furthermore, our experiments provided a potential explanation for the inconsistent results from earlier studies: our BiFC experiments showed that the interaction properties of AtSEOa and AtSEOb are influenced by the fused reporter fragments, as also reported for the assembly properties of NtSEO proteins fused to YFP (Ernst et al., 2012). The recombinant AtSEO fusion proteins monitored in sieve elements passing the sieve pores were therefore likely to be constrained in their interaction properties, which might have led to a reduced sealing capacity.

Although the *Arabidopsis* exudation assay demonstrated the significant impact of AtSEO proteins on phloem translocation, the loss of photosynthate in P-protein-depleted *Arabidopsis* mutants was far less than previously reported in tobacco. This may reflect the different sieve tube conductivity of the two species and its impact on exudation (Thompson and Wolniak, 2008) but it is also possible that the impact of P-proteins in sieve tube sealing or their general sealing efficiency varies among different species. Sieve element and sieve plate anatomy (e.g., the number and size of pores) differ widely between species (Mullendore et al., 2010) and the response to injury may consequently involve differing mechanisms. Therefore, the detailed interplay between P-proteins, callose and potential additional sieve element components (such as sieve element plastids) in phloem wound sealing will certainly be the topic of interesting future studies.

## Conflict of Interest Statement

The authors declare that the research was conducted in the absence of any commercial or financial relationships that could be construed as a potential conflict of interest.
